# Degeneration of dopaminergic circuitry influences depressive symptoms in Lewy body disorders

**DOI:** 10.1111/bpa.12697

**Published:** 2019-01-29

**Authors:** Lina Patterson, Steven P. Rushton, Johannes Attems, Alan J. Thomas, Christopher M. Morris

**Affiliations:** ^1^ Alzheimer's Society Doctoral Training Centre Newcastle University, Campus for Ageing and Vitality Newcastle upon Tyne UK; ^2^ School of Biology Newcastle University, Ridley Building Newcastle upon Tyne UK; ^3^ Newcastle upon Tyne Hospitals NHS Foundation Trust Newcastle‐upon‐Tyne UK; ^4^ Gateshead Health NHS Foundation Trust, Queen Elizabeth Hospital Gateshead UK; ^5^ NIHR Biomedical Research Centre Newcastle, Biomedical Research Building, Newcastle University, Campus for Ageing and Vitality Newcastle upon Tyne UK

**Keywords:** dementia with Lewy bodies, depression, α‐synuclein, dopaminergic pathways

## Abstract

****Aims**:**

Depression is commonly observed even in prodromal stages of Lewy body disorders (LBD), and is associated with cognitive impairment and a faster rate of cognitive decline. Given the role of dopamine in the development of movement disorders, but also in motivation and reward, we investigated neurodegenerative pathology in dopaminergic circuitry in Parkinson's disease (PD), PD with dementia (PDD) and dementia with Lewy bodies (DLB) patients in relation to depressive symptoms.

****Methods**:**

α‐synuclein, hyperphosphorylated tau and amyloid‐beta pathology was assessed in 17 DLB, 14 PDD and 8 PD cases within striatal and midbrain subregions, with neuronal cell density assessed in substantia nigra and ventral tegmental area. Additionally, we used a structural equation modeling (SEM) approach to investigate the extent to which brain connectivity might influence the deposition of pathological proteins within dopaminergic pathways.

****Results**:**

A significantly higher α‐synuclein burden was observed in the substantia nigra (*P* = 0.006), ventral tegmental area (*P* = 0.011) and nucleus accumbens (*P* = 0.031) in LBD patients with depression. Significant negative correlations were observed between cell density in substantia nigra with Lewy body (LB) Braak stage (*P* = 0.013), whereas cell density in ventral tegmental area showed negative correlations with LB Braak stage (*P* = 0.026) and neurofibrillary tangle Braak stage (*P* = 0.007).

****Conclusions**:**

Dopaminergic α‐synuclein pathology appears to drive depression. Selective targeting of dopaminergic pathways may therefore provide symptomatic relief for depressive symptoms in LBD patients.

**Abbreviations**
Aβ: Amyloid‐beta
AD: Alzheimer's disease
α‐syn: Alpha‐synuclein
Cd: Caudate
CSDD: Cornell Scale for Depression in Dementia
DLB: Dementia with Lewy bodies
GDS: Geriatric Depression Scale
GPe: Globus pallidus externus
GPi: Globus pallidus internus
HPT: Hyperphosphorylated tau
LB: Lewy bodies
LBD: Lewy body disorders
LN: Lewy neurites
MMSE: Mini‐Mental State Examination
NAcc: Nucleus accumbens
NFT: Neurofibrillary tangles
PD: Parkinson's disease
PDD: Parkinson's disease with dementia
Pu: Putamen
SEM: Structural Equation Modelling
SN: Substantia nigra
SNRI: Serotonin–norepinephrine reuptake inhibitor
SSRI: Selective serotonin reuptake inhibitor
TCA: Tricyclic antidepressant
TeCA: Tetracyclic antidepressant
UPDRS: Unified Parkinson's Disease Rating Scale
VTA: Ventral tegmental area

## Introduction

Parkinson's disease (PD), PD with dementia (PDD) and dementia with Lewy bodies (DLB) are the most common Lewy body disorders (LBD) 91, sharing many clinical and pathological characteristics 59, 60. LBD are pathologically characterized by abnormal aggregation of misfolded α‐synuclein (α‐syn) protein, which is the major component of Lewy bodies (LB) and Lewy neurites (LN) 14, 58, the distribution of which is associated with motor and cognitive changes. Comorbid Alzheimer's disease (AD) pathology is commonly observed, particularly in DLB, in the form of extracellular amyloid plaques, of amyloid‐beta (Aβ) peptide, along with neurofibrillary tangles (NFT) and neuropil threads of hyperphosphorylated tau (HPT) protein 42.

The prevalence of neuropsychiatric symptoms in LBD is high 77, 81, 102, with depression being the most common prodromal psychiatric symptom in LBD patients, often preceding the onset of motor symptoms 57, 76, 88. Depression is also associated with cognitive impairment 29, 103 and faster rate of cognitive decline in LBD 12, 28. Monoaminergic deficits in depression are well‐established, such as serotonin (5‐HT), norepinephrine (NE) and dopamine 92. PD patients with depression show increased serotonin transporter binding in raphe and limbic regions 75, whereas decreased 5‐HT_1A_ receptor densities in limbic regions including insula, hippocampus and orbitofrontal cortex are seen 6. Selective serotonin reuptake inhibitors (SSRI) are the most commonly prescribed antidepressants in PD patients with depression, although their efficacy in treating depression in PD is not supported by placebo controlled clinical trials 90. In PD, there are noradrenergic deficits due to loss of neurons in the locus coeruleus (LC), with reductions in noradrenaline found in caudate, putamen and cortical regions 33, 34. These changes are suggested to be related to the presence of depression as an early indication of LBD, since staging of pathology suggests degeneration of the LC before SN degeneration 96. Some studies have shown a marked reduction in 5‐HT and NE levels in striatal, cortical and hippocampal regions in AD patients with depression 7, 17, 97, 98, indicating that deficits in these neurotransmitters may relate to depression in AD. The differences between depression in LBD and AD may lie in the neural substrates, with dopamine metabolism being the major neurochemical difference.

Dopamine deficiency in the nigrostriatal dopamine pathway caused by progressive α‐syn associated neurodegeneration and loss of dopaminergic neurons in the substantia nigra (SN) pars compacta results in dopamine depletion in the dorsal striatum and the development of motor symptoms in LBD 69. The mesolimbic dopamine pathway sends dopaminergic projections from the ventral tegmental area (VTA) within the midbrain to various cortical and subcortical regions including the nucleus accumbens (NAcc) in the ventral striatum. Consequently, the VTA plays a role in pathophysiology of mood disorders and cognitive deficits 18, 36, 63, 82, 86. The extensive interconnections of the insular cortex with basal ganglia and limbic system, also makes it a key integrator of cognitive and emotional processing 11, 32. Since anhedonia and loss of motivation are core symptoms of depression it is likely that dysfunction of dopaminergic circuitry is involved in mediating depressive behaviors 16, 95.

Dopamine depletion is observed in L‐dopa naïve PD and DLB cases, which correlates with reduction in attention and cognition 68, 70, 72, and this is likely to derive from changes to the dopaminergic VTA 74. Improved cognition and mood in PD is observed following L‐dopa therapy 56 and correlates with a basal failure to inactivate the default mode network 22. Structural changes to basal ganglia circuitry in LBD are minimal 15 or absent 101 suggesting that changes within specific nuclei may be the major contributors of psychiatric symptoms 2, 15, 20, 46. Some studies have suggested an association of major depression with neuropathological processes in AD, with a higher Aβ plaque and NFT burden within the hippocampus and cortical regions in depressed individuals 64, 78. Therefore, depressive prodromal symptoms in LBD may be directly linked to neuropathological changes in the brain.

Neurodegeneration along specific pathways are the neuropathological correlate of distinct clinical phenotypes and this is assumed to be due to prion‐like spread of misfolded proteins (eg, α‐syn and HPT) through anatomically interconnected brain regions 3, 21, 39, 53, 80, 89, 100. Nigrostriatal degeneration occurs early in PD and PDD, where distribution and spreading of α‐syn are closely correlated with clinical symptoms and disease progression, respectively 25, 89. Indeed, current pathological staging of LBD suggests spread of synuclein pathology from specific medullary nuclei to SN and then to allocortical and neocortical regions 14. Grafted fetal nigral neurons in PD patients show LB inclusions years after transplantation, suggesting that physical contacts between susceptible regions, axonal transport and trans‐synaptic transmission of misfolded and aggregated α‐syn might have a role in the pathogenesis and propagation of PD 48, 51. Therefore, specific clinical symptoms in LBD patients may be due to accumulation of misfolded proteins in different brain regions within specific anatomical pathways.

Since dopaminergic projections are involved in motor, cognitive and psychiatric deficits in LBD, changes in specific nuclei may relate to specific clinical symptoms. Therefore, we investigated how α‐syn, Aβ and HPT pathology affect nigrostriatal and mesolimbic dopaminergic circuitries in LBD in relation to depressive symptoms.

## Materials and Methods

### Post‐mortem tissue

All post‐mortem human brain tissue was obtained from Newcastle Brain Tissue Resource (NBTR), with ethical approval for the study granted by the Newcastle and North Tyneside NHS Research Ethics Committee. All participants had received detailed clinical assessments during life and had consented to the donation and use of their brain tissue for research purposes. Post‐mortem neuropathological assessment was performed according to standardized neuropathological diagnostic procedures, which together with clinical data was used to make a clinicopathological diagnosis 59.

Three disease groups were included in this study, 17 DLB, 14 PDD and 8 PD cases (supplementary Table S1). Of note, 4 DLB and 1 PDD case also fulfilled the neuropathological criteria for high AD neuropathological change 66 and can therefore be classified as neuropathologically mixed AD/DLB with a LBD clinical phenotype 99. Case selection for neuropathological analysis was based on the availability of clinical data, with sequential scores of Mini‐Mental State Examination (MMSE), the Unified Parkinson's Disease Rating Scale (UPDRS), as well as neuropsychiatric information. The clinical diagnosis of depression was made by consulting psychiatrist (supplementary Table S1). The inclusion criteria for depression diagnosis was made using the Cornell Scale for Depression in Dementia (CSDD) (≥8), as a validated rating scale for depression in dementia 5, or the Geriatric Depression Scale (GDS) (≥10), whiles less sensitive, shown to retain acceptable qualities when applied to a population of demented elderly patients 49. In the absence of clinical diagnosis of depression by a consulting psychiatrist, we used retrospective analysis of clinical records to verify the presence or absence of depression.

### Sample preparation

At autopsy, the brain weight and post‐mortem delay were recorded. The right hemisphere was fixed in 10% formalin. Following fixation, the hemisphere was cut into 7mm coronal slices then dissected into blocks and embedded in paraffin wax for neuropathological assessment. Paraffin embedded blocks selected for analysis corresponded to striatal and midbrain subregions, including the NAcc, caudate nucleus, anterior and posterior putamen, globus pallidus internus and externus, insula cortex, as well as SN and VTA at the level of the red nucleus. Six micrometer sections were immunostained with primary antibodies to α‐syn, HPT and Aβ following antigen retrieval according to optimized protocols (supplementary Table S3) 99. The midbrain sections were stained with Cresyl fast violet to assess cell density, with the images captured using 63x oil immersion. A modified stereological method was employed to determine the neuronal cell density 67, with values calculated as cells per mm^2^.

### Neuropathological assessment

For quantification of neuropathological lesions, section images were captured using a Zeiss Z1 microscope and MRc camera (Zeiss, Germany) coupled to a PC. The images were sampled using stereological methods, with the region of interest drawn at 1.25× magnification. Dissector boxes were placed in a uniform and unbiased way within the region of interest using stereology software (Stereologer, Chester, MD, USA), with 10–15 frames captured at 10× magnification per region of interest for densitometric analysis. The images were analyzed using Fiji Image J analysis software (Windows 64‐bit: https://fiji.sc) 87. The mean percentage area stained for each frame was determined using the Red‐Green‐Blue (RGB) thresholds, which were adjusted manually for each antibody to eliminate the detection of non‐specific staining. The mean percentage area stained per case in all the regions was calculated from the mean values obtained across all images taken 99.

### Statistical analysis

Statistical analysis was undertaken in SPSS Statistics version 23.0 and in R. Assuming normally distributed data and equal group sizes (n = 15 with/without depression) and 20% standard deviation of the mean of a specific measure, the study was powered (β = 0.8 at α = 0.05) to detect an effect size of 0.21. Distributional assumptions for outcome measures were assessed using the Shapiro–Wilk test. As the neuropathological data was not normally distributed, we used non‐parametric Mann–Whitney test for comparison of two groups and Kruskal–Wallis test for multiple groups. Trends in MMSE and UPDRS through time for different disease groups were investigated using linear mixed effect modeling. These models were fitted individually for MMSE and UPDRS measures in the nlme package in R. Cohen's effect (d′) was calculated as an estimate of change of rate in MMSE and UPDRS scores 10. Correlation analyses were carried out using Spearman's correlation coefficient ρ (rho). A canonical correspondence analysis was used to investigate the impacts of pathology, age, Braak NFT stage 13 and disease groups on the levels of protein deposition measured across all patients. This model was undertaken in the vegan package in R. We then created a conceptual model of the network of physical connectivity between different brain regions and investigated how levels of protein deposition were related across the connectivity network using Structural Equation Modeling (SEM). Since only 39 patients were available we used a Bayesian approach to investigate the extent of the connectivity network of protein deposition. The models were fitted in the Jags package in R.

## Results

### Demographics

A total of 39 cases were included in this study for the neuropathological assessment. No significant difference was found in the age (*P* = 0.101) or post‐mortem delay (*P* = 0.784) between different disease groups. The decline in MMSE scores with time was slower in PD patients compared to PDD and DLB (t = 6.490, *P* < 0.001), with no significant difference observed between PDD and DLB patients (t = 0.619, *P* = 0.54). There was no difference between UPDRS scores for PD and DLB (t = 0.256, *P* = 0.799), although scores were significantly higher in patients diagnosed with PDD (t = 3.332, *P* = 0.002) (supplementary Figure [Link], [Link]). NFT Braak stage was not a significant predictor of MMSE (t = 0.142, *P* = 0.887) or UPDRS scores (t = 0.480, *P* = 0.635).

### Clinical presentation

Within the cohort, 8 DLB, 9 PDD and 4 PD cases were diagnosed with depression. There was no significant difference in the age (*P* = 0.754), post‐mortem delay (*P* = 0.987), NFT Braak stage (*P* = 0.526) or LB Braak stage (*P* = 0.315) in cases with and without depression. LBD cases with and without depression also showed no differences in baseline (*P* = 0.272) or last (*P* = 0.688) MMSE scores, as well as baseline (*P* = 0.167) or last (*P* = 0.273) UPDRS scores. Sixty six percent of LBD cases with depression were using SSRI, 9% SNRI and 24% TCA/TeCA antidepressant medication.

### Neuropathology

**Figure 1:**
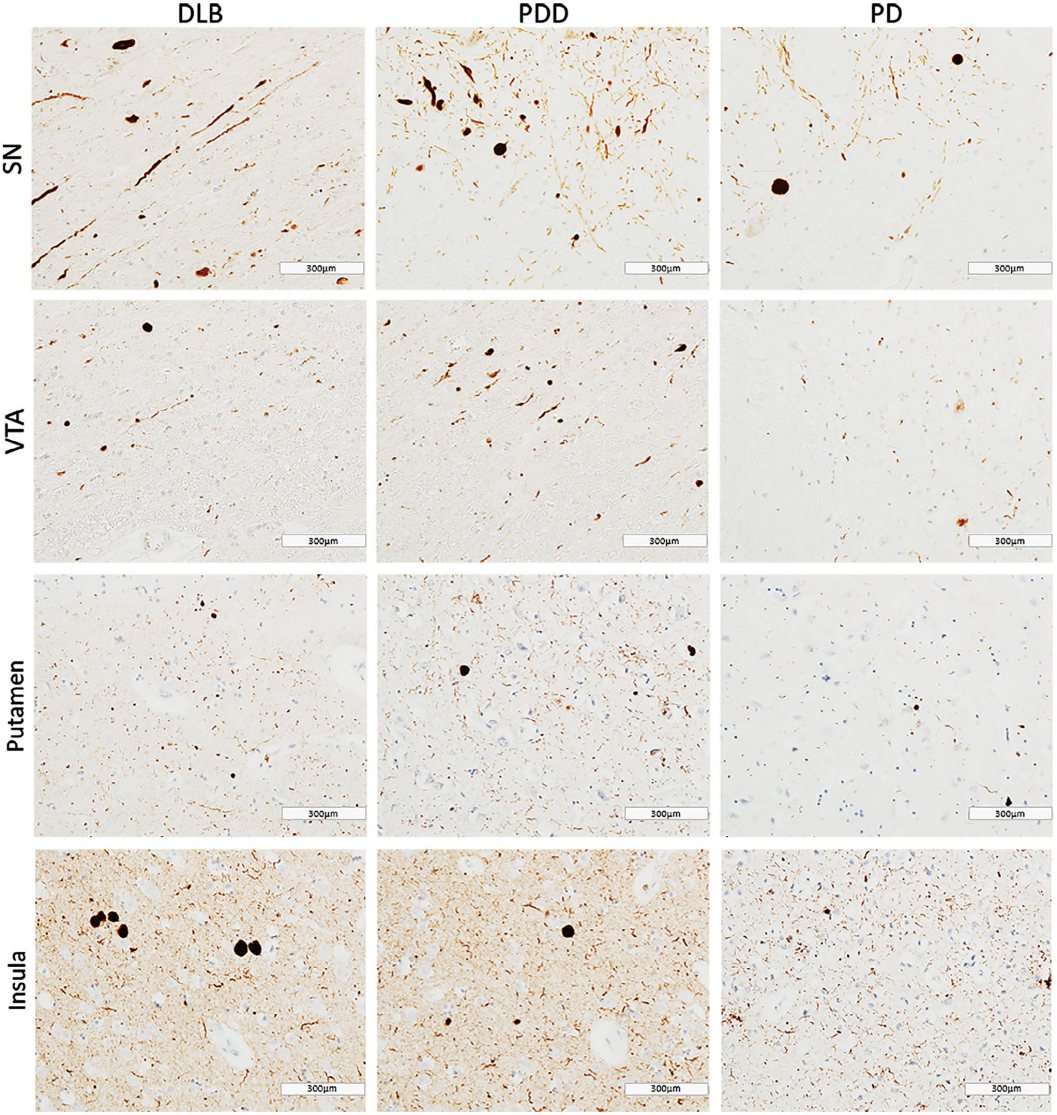
*α‐synuclein pathology distribution* **. **Photomicrographs of α‐synuclein pathology distribution in substantia nigra (SN), ventral tegmental area (VTA), putamen and insula in DLB (case #4, LB Braak stage 6), PDD (case #26, LB Braak stage 6) and PD (case #34, LB Braak stage 4). α‐synuclein immunohistochemistry with KM51 antibody, ×20. Scale bar 300 µm.

**Figure 2:**
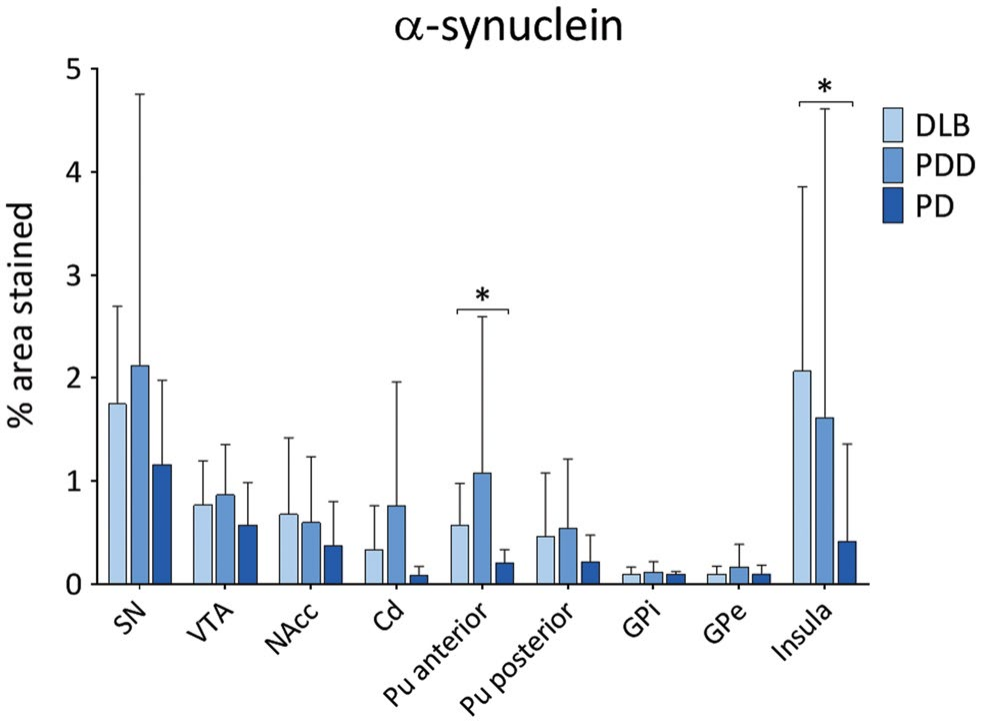
*Levels of α‐synuclein pathology in LBD* **. **α‐synuclein pathology (% area stained) was assessed in substantia nigra (SN), ventral tegmental area (VTA), nucleus accumbens (NAcc), caudate (Cd), anterior and posterior putamen (Pu), globus pallidus internus (GPi) and externus (GPe) and insula between DLB, PDD and PD groups. *P < 0.05, DLB vs. PD.

In general, PDD cases showed highest α‐syn burden across the regions of interest, with moderate to severe α‐syn pathology (LN and LB) observed in insula, SN, VTA and putamen (Figure 1). Alpha‐synuclein burden was significantly different in caudate (*H*(2) = 2.368, *P* = 0.046), anterior putamen (*H*(2) = 6.539, *P* = 0.038) and insula (*H*(2) = 8.391, *P* = 0.015) across disease groups. Paired comparison between groups showed higher α‐syn immunoreactivity in DLB cases compared to PD in anterior putamen (*H*(2) = 11.728, *P* = 0.041) and insula (*H*(2) = 13.383, *P* = 0.014). No significant difference was observed in α‐syn burden between DLB and PDD in midbrain and striatal subregions (Figure 2).

**Figure 3:**
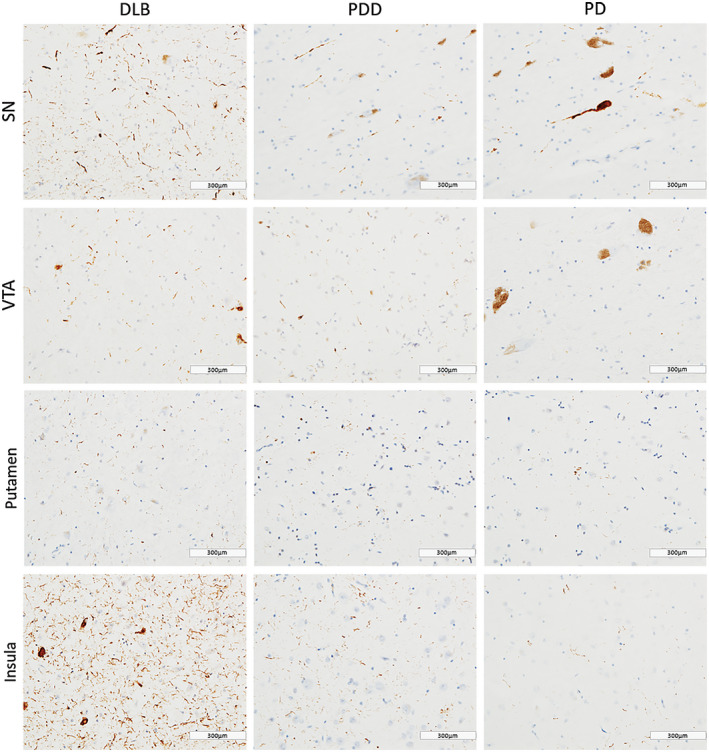
*Tau pathology distribution* **. **Photomicrographs of tau pathology distribution in substantia nigra (SN), ventral tegmental area (VTA), putamen and insula in DLB (case #4, NFT Braak stage 6), PDD (case #26, NFT Braak stage 6) and PD (case #34, NFT Braak stage 4). HPT immunohistochemistry with AT8 antibody, ×20. Scale bar 300 µm.

**Figure 4:**
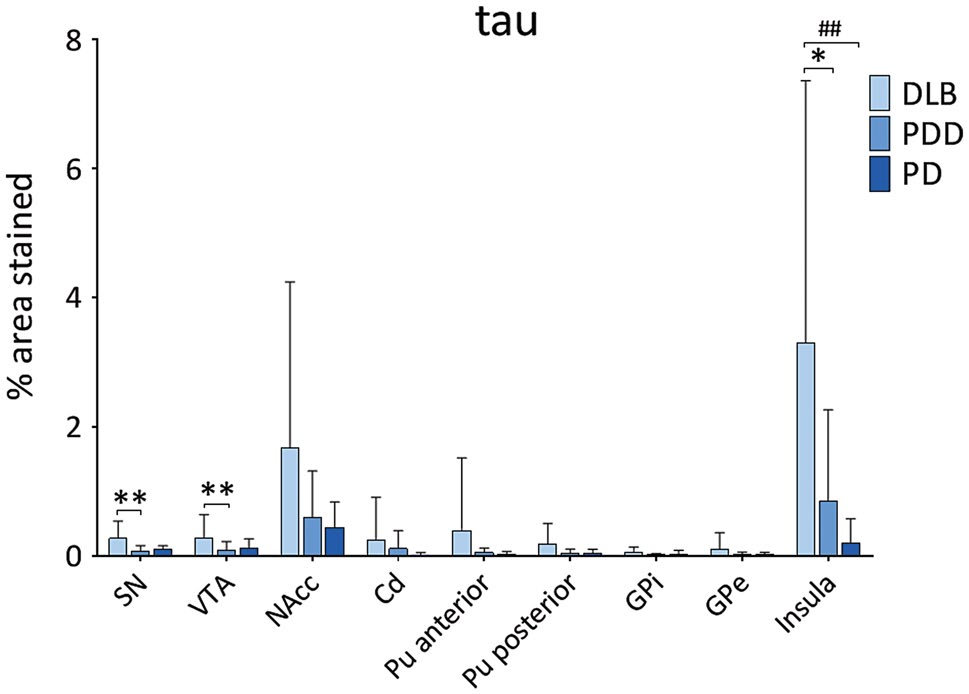
*Levels of tau pathology in LBD* **. **Tau pathology (% area stained) was assessed in substantia nigra (SN), ventral tegmental area (VTA), nucleus accumbens (NAcc), caudate (Cd), anterior and posterior putamen (Pu), globus pallidus internus (GPi) and externus (GPe) and insula between DLB, PDD and PD groups. *P < 0.05, DLB vs. PDD; **P < 0.01, DLB vs. PDD; ^##^ *P* < 0.01, DLB vs. PD.

DLB cases showed highest HPT burden across all regions (Figure 3). HPT burden was significantly different across disease groups in SN (*H*(2) = 10.374, *P* = 0.006), VTA (*H*(2) = 8.557, *P* = 0.014) and insula (*H*(2) = 11.080, *P* = 0.004). Paired analysis showed higher HPT burden in DLB compared to PDD cases in SN (*H*(2) = 13.055, *P* = 0.005), VTA (*H*(2) = 12.029, *P* = 0.010) and insula (*H*(2) = 10.576, *P* = 0.031), as well as in DLB compared to PD in insula (*H*(2) = 15.092, *P* = 0.006). No significant difference was observed in HPT burden between disease groups in any striatal or pallidal subregion (Figure 4).

**Figure 5:**
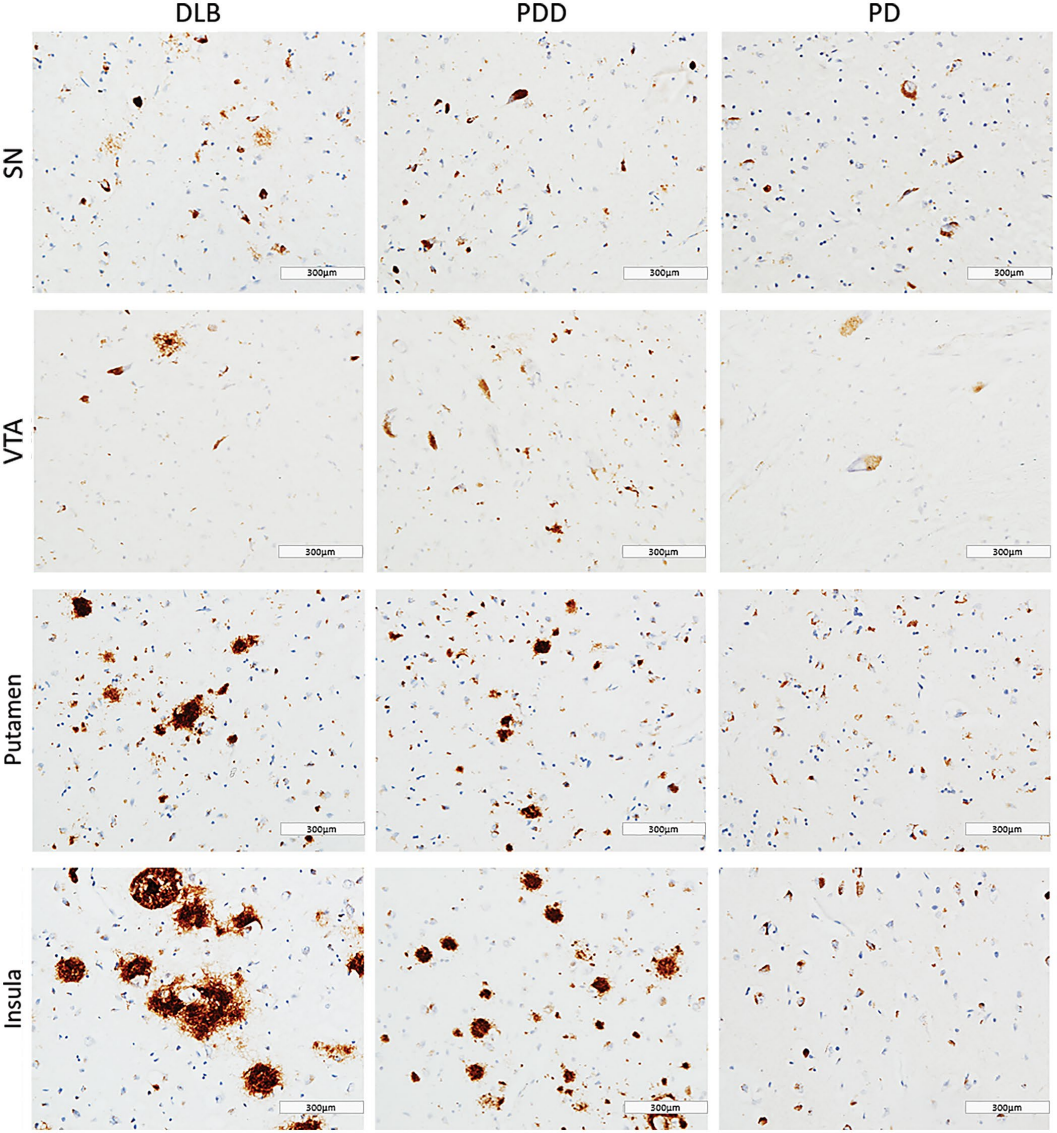
*Amyloid‐beta distribution* **. **Photomicrographs of Aβ pathology distribution in substantia nigra (SN), ventral tegmental area (VTA), putamen and insula in DLB (case #4), PDD (case #26) and PD (case #34). Aβ immunohistochemistry with 4G8 antibody, ×20. Scale bar 300 µm.

**Figure 6:**
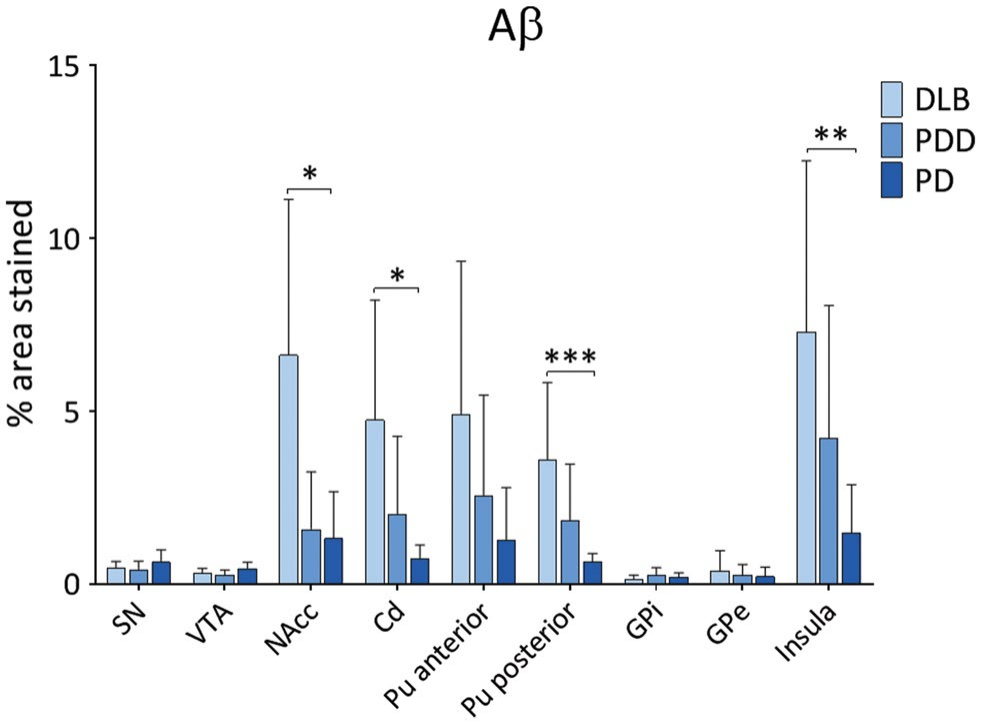
*Amyloid‐beta pathology in LBD* **. **Aβ pathology (% area stained) was assessed in substantia nigra (SN), ventral tegmental area (VTA), nucleus accumbens (NAcc), caudate (Cd), anterior and posterior putamen (Pu), globus pallidus internus (GPi) and externus (GPe) and insula between DLB, PDD and PD groups. *P < 0.05, DLB vs. PD; **P < 0.01, DLB vs. PD; ***P < 0.001, DLB vs. PD.

Diffuse Aβ plaques were observed in insula and putamen in DLB and PDD cases, whereas sparse Aβ deposits in SN and VTA (Figure 5). Aβ burden was significantly different in NAcc (*H*(2) = 7.890, *P* = 0.019), caudate (*H*(2) = 8.775, *P* = 0.012), anterior putamen (*H*(2) = 6.533, *P* = 0.038), posterior putamen (*H*(2) = 13.814, *P* = 0.001) and insula (*H*(2) = 9.876, *P* = 0.007) between disease groups. Paired analysis showed higher Aβ burden in DLB compared to PD in NAcc (*H*(2) = 11.143, *P* = 0.045), caudate (*H*(2) = 11.375, *P* = 0.030), posterior putamen (*H*(2) = 17.312, *p* = 0.001) and insula (*H*(2) = 14.368, *P* = 0.010) (Figure 6). No significant differences were observed in Aβ burden between DLB and PDD in midbrain or pallidal subregions.

**Figure 7:**
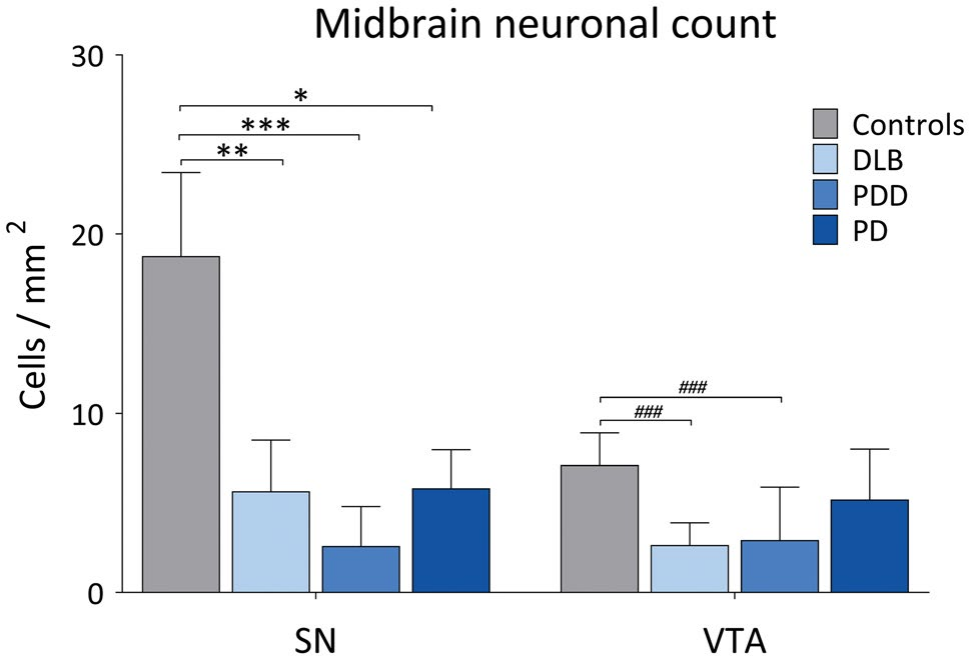
*Dopaminergic cell loss in the midbrain in LBD*. Pigmented dopaminergic neuron counts in substantia nigra (SN) and ventral tegmental area (VTA) (cells/mm^2^) in controls, DLB, PDD and PD cases. *P < 0.05, controls vs. PD; **P < 0.01, controls vs. DLB; ***P < 0.001, controls vs. PDD; ^###^ *P* < 0.001, controls vs. DLB and controls vs. PDD.

Pigmented dopaminergic neurons were counted in SN and VTA, with means taken across the groups, generating (cells/mm^2^) values. Neuronal count in SN was significantly higher in controls compared to DLB (*H*(3) = 20.25, *P* = 0.001), PDD (*H*(3) = 32.0, *P* < 0.001) and PD (*H*(3) = 19.21, *P* = 0.028). Neuronal density in VTA was significantly higher in controls compared to DLB (*H*(3) = 22.18, *P* < 0.001) and PDD (*H*(3) = 22.16, *P* < 0.001), with no significant difference observed between controls and PD (Figure 7), although a mean reduction of around 20% was observed. Cell density in SN showed significant negative correlation with LB Braak stage (*r* = −0.415, *P* = 0.013), whereas cell density in VTA showed negative correlations with LB Braak stage (*r* = −0.360, *P* = 0.026) and NFT Braak stage (*r* = −0.429, *P* = 0.007).

### Clinical and pathological correlates

The rate of change in MMSE scores in LBD cases showed significant negative correlations with α‐syn burden in anterior putamen (*r* = −0.329, *P* = 0.046), insula (*r* = −0.455, *P* = 0.008) and LB Braak stage (*r* = −0.384, *P* = 0.025). Negative correlations were also observed between cognitive decline and tau pathology in anterior putamen (*r* = −0.455, *P* = 0.008), insula (*r* = −0.503, *P* = 0.002) and NFT Braak stage (*r* = −0.370, *P* = 0.031), whereas Aβ burden with NAcc (*r* = −0.428, *P* = 0.018), caudate (*r* = −0.399, *P* = 0.017), anterior putamen (*r* = −0.420, *P* = 0.011), posterior putamen (*r* = −0.408, *P* = 0.018) and insula (*r* = −0.411, *P* = 0.019). No correlations were observed between pathological burden and UPDRS rate of change.

**Figure 8:**
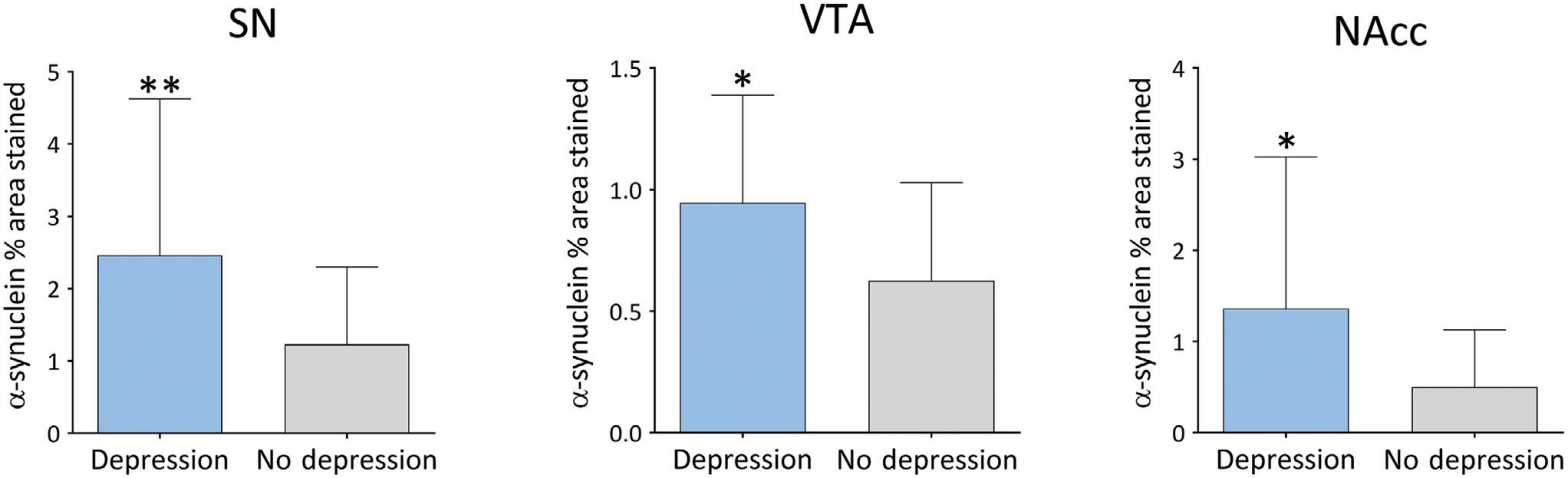
*α‐synuclein burden in depressed vs. non‐depressed LBD cases* **. **Significant pathological changes (% area stained) in depressed vs. nondepressed LBD cases. Significance levels set at *P < 0.05, **P < 0.01 and ***P < 0.001.

LBD cases diagnosed with depression during life showed significantly higher α‐syn burden in SN (*U* = 2.719, *P* = 0.006), VTA (*U* = 2.521, *P* = 0.011) and NAcc (*U *= 2.155, *P *= 0.031; Figure 8), whereas no significant differences were observed between LBD cases with depression and pathological burden of HPT and Aβ in striatal or midbrain subregions.

### Canonical correspondence analysis

**Figure 9:**
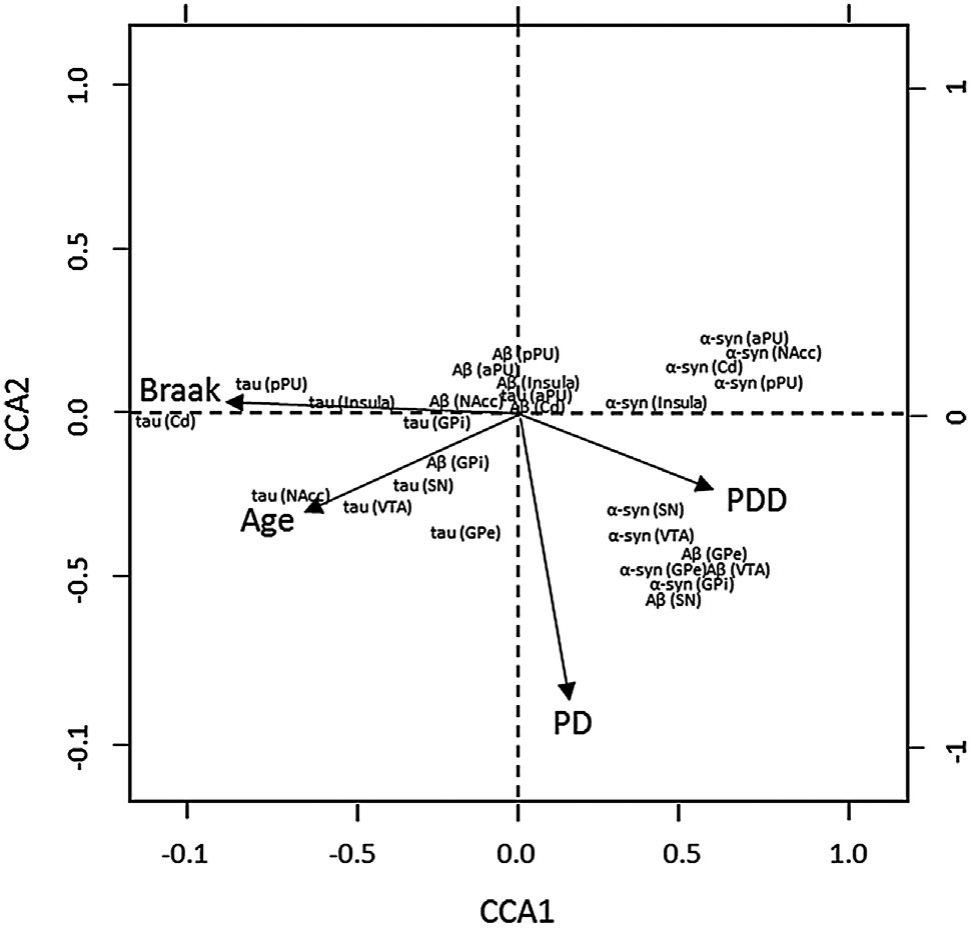
*Canonical correspondence analysis* **. **Pathological protein burden across brain regions in relation to age, Braak NFT stage and disease group. Note, DLB is the reference in this plot against which PD and PDD are compared.

Canonical correspondence analysis was used to explore relationships between pathological protein burden across brain regions in relation to age, NFT Braak stage and disease group. The major trend (ie, Axis 1) was related to high levels of tau proteins (negative Axis 1) through to high levels of α‐syn (high positive axis 1). The disorders clearly separated PDD (with high α‐syn deposition) on the positive side and DLB and PD to the negative side. PD cases were more associated with negative scores on the second axis. Age and Braak NFT stage were also negatively associated with the first axis indicating that older patients tended to be found with higher levels of HPT protein deposition, indicative of the separation of DLB and PDD (Figure 9). Since different parts of the brain are interconnected, it is likely that the measures of the proteins in different brain region are not independent of each other. Therefore, it is likely that the presence of proteins in one compartment will be dependent on those in the brain to which they are directly connected.

### Pathological spread modeling

We created a conceptual model of connectivity based on known major pathways of neuronal linkage between the regions being studied (supplementary Table S4), and employed measures of protein deposition in these areas as response variables in a SEM approach to investigate the extent to which brain connectivity might influence the deposition of pathological proteins and development of symptoms. Density plots for the coefficients for three models are shown in supplementary Figures [Link], [Link], [Link].

**Figure 10:**
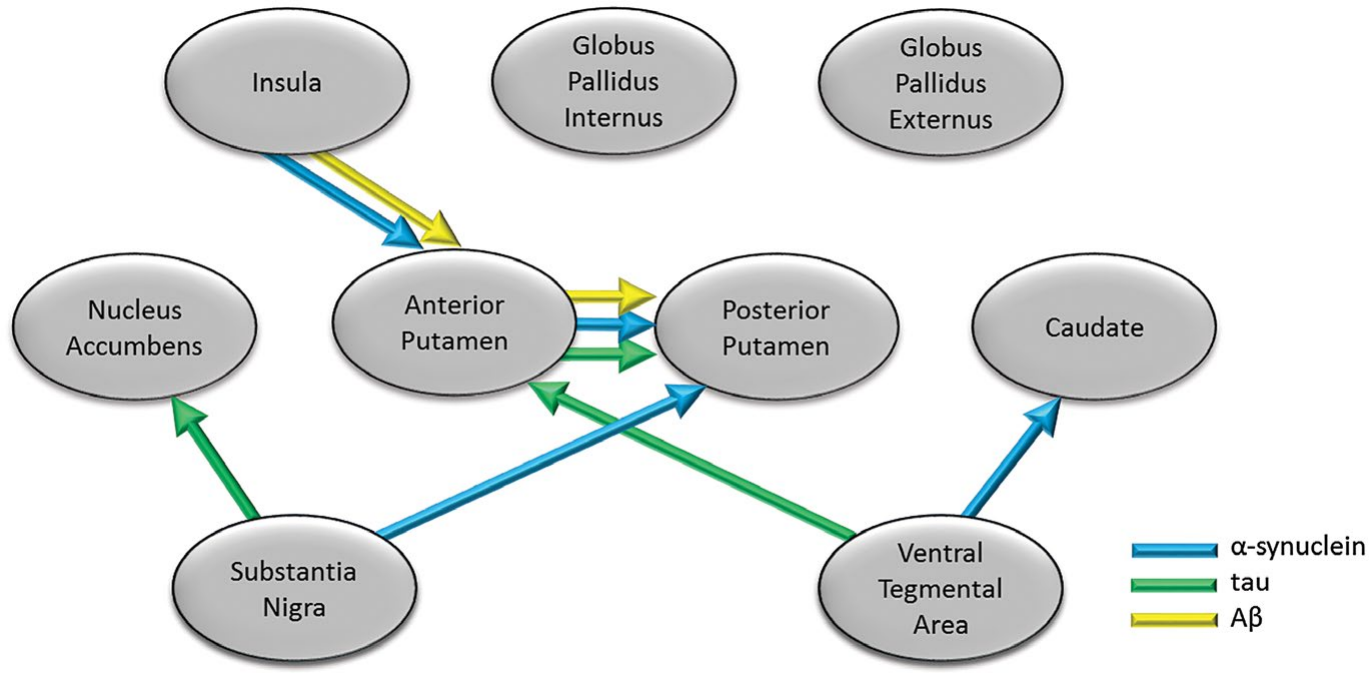
*Modeling of pathology spread in LBD* **. **Structural Equation Model suggesting significant spread along pathways between brain regions based on levels of protein deposition in each region. Arrows indicate significant linkages between brain regions for α‐synuclein, tau and Aβ, where the levels of protein in one region (end of arrow) were significantly related to levels in the other region from which the arrow starts (in this case assumed to be source).

Significant linkages between brain regions were observed for each protein (Figure 10). For α‐syn there were significant associations between levels of protein in VTA with anterior caudate. For SN, linkages with posterior putamen and α‐syn were seen. Anterior putamen with posterior putamen, as well as insula cortex with anterior putamen also showed linkage. Weaker association trending on significance was observed between α‐syn level in SN with nucleus accumbens. HPT burden in SN was strongly linked to HPT burden in the nucleus accumbens. The anterior putamen showed linkage with posterior putamen, while HPT burden in VTA showed strongest linkage with the anterior putamen. Weaker linkages trending on significance were observed between SN with anterior putamen, as well as VTA with nucleus accumbens. In the case of Aβ there were significant linkages between levels of protein in the insula with anterior putamen, as well as anterior putamen with posterior putamen. Weaker linkages trending on significance for Aβ were observed in posterior putamen with globus pallidus internus, as well as globus pallidus internus with externus.

## Discussion

Depression in LBD is common 1, 8, 77, causing major loss in quality of life 61, 62, however, the underlying systems that contribute to depression are unclear. Given the success of dopamine therapy in PD in reducing depressive symptoms 30, 40, 84, 85, 105, identifying those systems that contribute to depression across the LBD spectrum may have similar therapeutic success. We therefore investigated dopaminergic system pathology in LBD in relation to depression.

Pathological burden of α‐syn was significantly higher in dorsal striatum and insula in DLB compared to PD cases, consistent with other studies 37, 43, 45. LBD cases with depression showed significantly higher α‐syn levels in SN, VTA and NAcc, suggesting that the levels of α‐syn pathology may be one of the main factors driving these symptoms. An increase in subcortical and cortical LB pathology has been observed in major depressive disorder 41, 94, as well as in amygdala in AD cases with a history of depression 52. Alpha‐synuclein interaction with dopamine metabolism 31 and transmission 73, 107 may have important implications not only in neuronal loss and motor symptoms 9, 71, but also through development of depressive symptoms in LBD. Dysfunction in striatal connectivity with limbic and cortical areas is suggested to underpin many neuropsychiatric symptoms in LBD 44, supported by the strong interaction of mesolimbic circuits with emotional processing 54. Our finding of an association between α‐synuclein pathology and depression in LBD strongly implicates spread of pathology from mesolimbic and mesostriatal dopaminergic neurons, which is supported by our finding of associations of α‐synuclein spread through SEM (Figure 6). Based on our conceptual model of progression of neurodegeneration within dopaminergic pathways, this continued progression of symptoms may be underpinned by the neuronal connectivity in the brain and the spread of specific pathologies. A significant relationship was observed for α‐syn spread from VTA to caudate, SN to putamen, as well as spread from the insula cortex to putamen. This suggested spread of α‐syn from brainstem to both motor and non‐motor parts of the striatum may indicate that not only mesolimbic, but also nigrostriatal dopaminergic circuits are needed in depression 4, 27.

DLB cases had higher HPT pathological burden in all striatal subregions and insula compared to other groups, as well as significantly higher burden in VTA and SN compared to PDD. Other studies have demonstrated significantly higher HPT burden within striatal regions in DLB compared to PDD, perhaps in line with a generally higher NFT Braak scores in DLB 38, 45. Some studies have shown an association between AD cases with a lifetime history of depression and higher burden of NFT pathology in hippocampus 78, 79, as well as a positive correlation between depressive states and density of neuritic plaques in patients with dementia 65. Other studies have however shown an association of depressive symptoms in AD to be independent of cortical and brainstem plaques and tangles 93, 104. In this study, we found no association between depression and HPT pathological burden within nigrostriatal and mesolimbic dopaminergic pathways in LBD cases. HPT levels showed strong linkages from both SN and VTA to dorsal and ventral striatum in relation to potential spread of pathology using SEM (Figure 6), which may suggest that higher HPT burden within dopaminergic pathways contributes to faster cognitive decline.

In line with data from PiB imaging studies 35, we also observed higher striatal Aβ deposition in DLB cases compared to PDD and PD. Striatal Aβ burden is suggested to be a strong correlate of dementia in PD 47, with differences in striatal Aβ deposition contributing to the timing of dementia relative to parkinsonism in DLB and PDD 45, potentially as a consequence of developing AD pathology. In this study, no relationship was observed between Aβ burden in dopaminergic circuitry and depression. While the potential role of Aβ in the pathophysiology of depression is unclear, some studies have shown an increase in binding using PET imaging in cortical regions in late‐life depression 50, 106, while others failed to associate PET amyloid burden with depressive symptoms 19, 24, 55. Aβ showed a different pattern of pathology spread, primarily driven by inputs from the insula to anterior putamen, and also spread from anterior putamen to posterior putamen, which may indicate more cortically driven spread of Aβ pathology linked to cognition.

Overall, we observed clear time trends in MMSE and UPDRS scores, which were independent of age at onset or death and may therefore represent the continuing dysfunction and potentially pathological spread of changes within the CNS in LBD. The trends in cognitive decline were similar in DLB and PDD patients, but slower in PD cases, while decline in motor function with time was much greater in PDD patients than in PD or DLB. Significant negative correlations were observed between HPT and Aβ burden in dopaminergic nuclei, as well as limbic brain regions in LDB cases with faster rate of cognitive decline, which may suggest that AD pathology plays an important role in cognitive dysfunction in LBD, and progression of AD pathology may therefore relate to progression of symptoms.

Previous studies have shown α‐syn pathological burden to be associated with nigral neuronal loss 23, 26, 83. In this study, neuronal loss in SN was greater with longer disease duration and a higher LB Braak stage, which may explain more severe neuronal loss in PDD cases. A higher burden of coexisting α‐syn and AD type pathology in DLB and PDD cases may suggest increased neuronal vulnerability in the VTA.

These findings highlight the interaction between different types of pathology, which differentially affect brain structures within dopaminergic pathways, giving rise to certain clinical phenotypes. Dopaminergic α‐syn pathology appears to drive depression, therefore, targeting these specific pathways and mechanisms may provide relief from depressive symptoms in LBD patients.

All the cases in this study were well clinically characterized, with detailed neuropsychiatric information, as well as baseline and follow‐up motor and cognitive assessments. There are however, certain caveats to the study findings. While the prevalence of other psychiatric symptoms, such as visual hallucinations and REM sleep disorder, were not significantly different between depressed and non‐depressed cases due to the high prevalence of these symptoms in the study group, 33% of depressed patients also had concomitant anxiety symptoms (χ^2^ = 0.056), which may need to be taken into account in future larger studies. Further, the classification of major depressive disorder was performed using a number of available sources. Although ideally cases would have been defined using a unified clinical score for depression in our LBD cohort, the different measures still have suitability for detecting depression in older populations. Our study has naturally focused on the dopaminergic system in LBD and the results suggest dopaminergic changes are a driver of depression in LBD. However, given the relatively small number of cases in this study, replication will be necessary in larger studies, and we cannot rule out noradrenergic or serotonergic involvement in the pathogenesis of depression in LBD.

## Author contributions

LP collected the data, performed the analysis and interpretation, and wrote the manuscript. SPR performed canonical correspondence analysis and Structural Equation Modelling. JA undertook neuropathological diagnosis of cases used in the study and revisions of the manuscript. AJT provided clinical diagnosis and interpretation of the results and contributed to writing of the manuscript. CMM, AJT conceived and designed the study, provided supervision and funding, interpreted the data and revised the manuscript.

## Supporting information


**Figure S1**. Trends in MMES scores in DLB, PDD and PD patients through time.

**Figure S2**. Trends in UPDRS scores for DLB, PDD and PD patients through time.

**Figure S3**. Density plot for the SEM coefficients for the full model for deposition of α‐synuclein. Distributions for the coefficients beta(2), beta(5), beta(6) and beta(8) do not include zero, suggesting significant pathways between the two brain compartments.

**Figure S4**. Density plot for the SEM coefficients for the full model for deposition of tau protein. Distributions for the coefficients beta(3), beta(8) and beta(9) do not include zero suggesting significant pathways between the two brain compartments.

**Figure S5**. Density plot for the SEM coefficients for the full model for deposition of amyloid beta. Distributions for the coefficients beta(5) and beta(8) suggesting significant pathways between the two brain compartments.

**Table S1**. Demographic and neuropathological characteristics for post‐mortem cohort. PM delay refers to the time interval between death and fixation. McKeith criteria refers to Lewy body pathology. Braak stage, Braak NFT pathology stage. CERAD, consortium to establish a registry of AD. MMSE ‐ Mini‐Mental State Examination. UPDRS ‐ Unified Parkinson's Disease Rating Scale.

**Table S2**. Clinical characteristics for post‐mortem cohort. CSDD ‐ Cornell Scale for Depression in Dementia; GDS ‐ Geriatric Depression Scale; SNRI ‐ Serotonin–norepinephrine reuptake inhibitor; SSRI ‐ Selective serotonin reuptake inhibitor; TCA ‐ Tricyclic antidepressant; TeCA ‐ Tetracyclic antidepressant.

**Table S3**. Antibodies used for neuropathological analysis.

**Table S4**. Modeled links.

## References

[1] Aarsland D , Ballard C , Larsen JP , McKeith I (2001) A comparative study of psychiatric symptoms in dementia with Lewy bodies and Parkinson's disease with and without dementia. Int J Geriatr Psych 16:528–536. 10.1002/gps.389 11376470

[2] Agosta F , Canu E , Stefanova E , Sarro L , Tomic A , Spica V *et al* (2013) Mild cognitive impairment in Parkinson's disease is associated with a distributed pattern of brain white matter damage. Hum Brain Mapp 35:1921–1929. 23843285 10.1002/hbm.22302 PMC6869219

[3] Ahmed RM , Devenney EM , Irish M , Ittner A , Naismith S , Ittner LM *et al* (2016) Neuronal network disintegration: common pathways linking neurodegenerative diseases. J Neurol Neurosurg Psychiatry 87:1234–1241. 27172939 10.1136/jnnp-2014-308350 PMC5099318

[4] Alexander GE , Crutcher MD , DeLong MR (1990) Basal ganglia‐thalamocortical circuits: parallel substrates for motor, oculomotor, “prefrontal” and “limbic” functions. Prog Brain Res 85:119–146. 2094891

[5] Alexopoulos GS , Abrams RC , Young RC , Shamoian CA (1988) Cornell scale for depression in Dementia. Biol Psychiatry 23:271–284. 3337862 10.1016/0006-3223(88)90038-8

[6] Ballanger B , Klinger H , Eche J , Lerond J , Vallet AE , Le Bars D *et al* (2012) Role of serotonergic 1A receptor dysfunction in depression associated with parkinson's disease. Movement Disord 27:84–89. 21994070 10.1002/mds.23895

[7] Ballard C , Johnson M , Piggott M , Perry R , O'Brien J , Rowan E *et al* (2002) A positive association between 5HT re‐uptake binding sites and depression in dementia with Lewy bodies. J Affect Disorders 69:219–223. 12103469 10.1016/s0165-0327(00)00375-x

[8] Ballard CG , Jacoby R , Del Ser T , Khan MN , Munoz DG , Holmes C *et al* (2004) Neuropathological substrates of psychiatric symptoms in prospectively studied patients with autopsy‐confirmed dementia with lewy bodies. Am J Psychiatry 161:843–849. 15121649 10.1176/appi.ajp.161.5.843

[9] Bennett MC (2005) The role of alpha‐synuclein in neurodegenerative diseases. Pharmacol Ther 105:311–331. 15737408 10.1016/j.pharmthera.2004.10.010

[10] Biundo R , Weis L , Bostantjopoulou S , Stefanova E , Falup‐Pecurariu C , Kramberger MG , Geurtsen GJ *et al* (2016) MMSE and MoCA in Parkinson's disease and dementia with Lewy bodies: a multicenter 1‐year follow‐up study. J Neural Transm (Vienna) 123:431–438. 26852137 10.1007/s00702-016-1517-6 PMC4820017

[11] Blanc F , Noblet V , Philippi N , Cretin B , Foucher J , Armspach JP *et al* (2014) Right anterior insula: core region of hallucinations in cognitive neurodegenerative diseases. PLoS ONE 9:e114774. 25479196 10.1371/journal.pone.0114774 PMC4257732

[12] Boot BP , Orr CF , Ahlskog JE , Ferman TJ , Roberts R , Pankratz VS *et al* (2013) Risk factors for dementia with Lewy bodies: a case‐control study. Neurology 81:833–840. 23892702 10.1212/WNL.0b013e3182a2cbd1 PMC3908463

[13] Braak H , Alafuzoff I , Arzberger T , Kretzschmar H , DelTredici K (2006) Staging of Alzheimer disease‐associated neurofibrillary pathology using paraffin sections and immunocytochemistry. Acta Neuropathologica 112:389–404. 16906426 10.1007/s00401-006-0127-z PMC3906709

[14] Braak H , Del Tredici K , Bratzke H , Hamm‐Clement J , Sandmann‐Keil D , Rub U (2002) Staging of the intracerebral inclusion body pathology associated with idiopathic Parkinson's disease (preclinical and clinical stages). J Neurol 249(Suppl 3):III/1‐5. 10.1007/s00415-002-1301-4 12528692

[15] Burton EJ , McKeith IG , Burn DJ , Williams ED , O'Brien JT (2004) Cerebral atrophy in Parkinson's disease with and without dementia: a comparison with Alzheimer's disease, dementia with Lewy bodies and controls. Brain 127(Pt 4):791–800. 14749292 10.1093/brain/awh088

[16] Chaudhury D , Walsh JJ , Friedman AK , Juarez B , Ku SM , Koo JW *et al* (2013) Rapid regulation of depression‐related behaviours by control of midbrain dopamine neurons. Nature 493:532‐+. 23235832 10.1038/nature11713 PMC3554860

[17] Chen CP , Alder JT , Bowen DM , Esiri MM , McDonald B , Hope T *et al* (1996) Presynaptic serotonergic markers in community‐acquired cases of Alzheimer's disease: correlations with depression and neuroleptic medication. J Neurochem 66:1592–1598. 8627315 10.1046/j.1471-4159.1996.66041592.x

[18] Choi EY , Yeo BT , Buckner RL (2012) The organization of the human striatum estimated by intrinsic functional connectivity. J Neurophysiol 108:2242–2263. 22832566 10.1152/jn.00270.2012 PMC3545026

[19] Chung JK , Plitman E , Nakajima S , Chow TW , Chakravarty MM , Caravaggio F *et al* (2016) Lifetime history of depression predicts increased amyloid‐beta accumulation in patients with mild cognitive impairment. J Alzheimers Dis 49:1189–1190. 26756327 10.3233/JAD-159007

[20] Cousins DA , Burton EJ , Burn D , Gholkar A , McKeith IG , O'Brien JT (2003) Atrophy of the putamen in dementia with Lewy bodies but not Alzheimer's disease: an MRI study. Neurology 61:1191–1195. 14610119 10.1212/01.wnl.0000091889.20347.30

[21] Danzer KM , Kranich LR , Ruf WP , Cagsal‐Getkin O , Winslow AR , Zhu L *et al* (2012) Exosomal cell‐to‐cell transmission of alpha synuclein oligomers. Mol Neurodegener 7:42. 22920859 10.1186/1750-1326-7-42 PMC3483256

[22] Delaveau P , Salgado‐Pineda P , Fossati P , Witjas T , Azulay JP , Blin O (2010) Dopaminergic modulation of the default mode network in Parkinson's disease. Eur Neuropsychopharmacol 20:784–792. 20674286 10.1016/j.euroneuro.2010.07.001

[23] Dijkstra AA , Voorn P , Berendse HW , Groenewegen HJ , Netherlands Brain B , Rozemuller AJ , van de Berg WD (2014) Stage‐dependent nigral neuronal loss in incidental Lewy body and Parkinson's disease. Mov Disord 29:1244–1251. 24996051 10.1002/mds.25952

[24] Donovan NJ , Hsu DC , Dagley AS , Schultz AP , Amariglio RE , Mormino EC *et al* (2015) Depressive symptoms and biomarkers of alzheimer's disease in cognitively normal older adults. J Alzheimers Dis 46:63–73. 25697700 10.3233/JAD-142940 PMC4544638

[25] Fearnley JM , Lees AJ (1990) Striatonigral Degeneration—a Clinicopathological Study. Brain J Neurol 113:1823–1842. 10.1093/brain/113.6.1823 2276046

[26] Fearnley JM , Lees AJ (1991) Ageing and Parkinson's disease: substantia nigra regional selectivity. Brain 114(Pt 5):2283–2301. 1933245 10.1093/brain/114.5.2283

[27] Frisina PG , Haroutunian V , Libow LS (2009) The neuropathological basis for depression in Parkinson's disease. Parkinsonism Relat Disord 15:144–148. 18571456 10.1016/j.parkreldis.2008.04.038 PMC3071242

[28] Fritze F , Ehrt U , Hortobagyi T , Ballard C , Aarsland D (2011) Depressive symptoms in Alzheimer's disease and lewy body dementia: a one‐year follow‐up study. Dement Geriatr Cogn Disord 32:143–149. 21986003 10.1159/000332016

[29] Fujishiro H , Nakamura S , Sato K , Iseki E (2015) Prodromal dementia with Lewy bodies. Geriatr Gerontol Int 15:817–826. 25690399 10.1111/ggi.12466

[30] Funkiewiez A , Ardouin C , Cools R , Krack P , Fraix V , Batir A *et al* (2006) Effects of levodopa and subthalamic nucleus stimulation on cognitive and affective functioning in Parkinson's disease. Mov Disord Off J Mov Disord Soc 21:1656–1662. 10.1002/mds.21029 16830317

[31] Galvin JE (2006) Interaction of alpha‐synuclein and dopamine metabolites in the pathogenesis of Parkinson's disease: a case for the selective vulnerability of the substantia nigra. Acta Neuropathol 112:115–126. 16791599 10.1007/s00401-006-0096-2

[32] Ghaziri J , Tucholka A , Girard G , Houde JC , Boucher O , Gilbert G *et al* (2015) The corticocortical structural connectivity of the human insula. Cereb Cortex 27:1216–1228. 10.1093/cercor/bhv308 26683170

[33] Gibb WR (1992) Neuropathology of Parkinson's disease and related syndromes. Neurol Clin 10:361–376. 1584179

[34] Goldstein DS , Sullivan P , Holmes C , Kopin IJ , Basile MJ , Mash DC (2011) Catechols in post‐mortem brain of patients with Parkinson disease. Eur J Neurol 18:703–710. 21073636 10.1111/j.1468-1331.2010.03246.x PMC4580229

[35] Gomperts SN , Rentz DM , Moran E , Becker JA , Locascio JJ , Klunk WE *et al* (2008) Imaging amyloid deposition in Lewy body diseases. Neurology 71:903–910. 18794492 10.1212/01.wnl.0000326146.60732.d6 PMC2637553

[36] Groenewegen HJ , Room P , Witter MP , Lohman AHM (1982) Cortical afferents of the nucleus accumbens in the cat, studied with anterograde and retrograde transport techniques. Neuroscience 7:977–996. 7099426 10.1016/0306-4522(82)90055-0

[37] Halliday GM , Holton JL , Revesz T , Dickson DW (2011) Neuropathology underlying clinical variability in patients with synucleinopathies. Acta Neuropathol 122:187–204. 21720849 10.1007/s00401-011-0852-9

[38] Halliday GM , Song YJ , Harding AJ (2011) Striatal beta‐amyloid in dementia with Lewy bodies but not Parkinson's disease. J Neural Transm (Vienna) 118:713–719. 21479514 10.1007/s00702-011-0641-6

[39] Hansen C , Angot E , Bergstrom AL , Steiner JA , Pieri L , Paul G *et al* (2011) alpha‐Synuclein propagates from mouse brain to grafted dopaminergic neurons and seeds aggregation in cultured human cells. J Clin Invest 121:715–725. 21245577 10.1172/JCI43366 PMC3026723

[40] Harada T , Ishizaki F , Horie N , Nitta Y , Yamada T , Sasaki T *et al* (2011) New dopamine agonist pramipexole improves parkinsonism and depression in Parkinson's disease. Hiroshima J Med Sci 60:79–82. 22389951

[41] Iritani S , Tsuchiya K , Arai T , Akiyama H , Ikeda K (2008) An atypical autopsy case of Lewy body disease with clinically diagnosed major depression: a clinical, radiological and pathological study. Neuropathology 28:652–659. 18410279 10.1111/j.1440-1789.2008.00905.x

[42] Irwin DJ , Grossman M , Weintraub D , Hurtig HI , Duda JE , Xie SX *et al* (2017) Neuropathological and genetic correlates of survival and dementia onset in synucleinopathies: a retrospective analysis. Lancet Neurol 16:55–65. 27979356 10.1016/S1474-4422(16)30291-5 PMC5181646

[43] Irwin DJ , White MT , Toledo JB , Xie SX , Robinson JL , Van Deerlin V *et al* (2012) Neuropathologic substrates of Parkinson disease dementia. Ann Neurol 72:587–598. 23037886 10.1002/ana.23659 PMC3484250

[44] Ishii T , Sawamoto N , Tabu H , Kawashima H , Okada T , Togashi K *et al* (2016) Altered striatal circuits underlie characteristic personality traits in Parkinson's disease. J Neurol 263:1828–1839. 27334907 10.1007/s00415-016-8206-0

[45] Jellinger KA , Attems J (2006) Does striatal pathology distinguish Parkinson disease with dementia and dementia with Lewy bodies? Acta Neuropathol 112:253–260. 16804711 10.1007/s00401-006-0088-2

[46] Jokinen P , Bruck A , Aalto S , Forsback S , Parkkola R , Rinne JO (2009) Impaired cognitive performance in Parkinson's disease is related to caudate dopaminergic hypofunction and hippocampal atrophy. Parkinsonism Relat Disord 15:88–93. 18434233 10.1016/j.parkreldis.2008.03.005

[47] Kalaitzakis ME , Graeber MB , Gentleman SM , Pearce RK (2008) Striatal beta‐amyloid deposition in Parkinson disease with dementia. J Neuropathol Exp Neurol 67:155–161. 18219254 10.1097/NEN.0b013e31816362aa

[48] Kordower JH , Chu YP , Hauser RA , Freeman TB , Olanow CW (2008) Lewy body‐like pathology in long‐term embryonic nigral transplants in Parkinson's disease. Nat Med 14:504–506. 18391962 10.1038/nm1747

[49] Korner A , Lauritzen L , Abelskov K , Gulmann N , Brodersen AM , Wedervang‐Jensen T , Kjeldgaard KM (2006) The geriatric depression scale and the Cornell Scale for Depression in Dementia. A validity study. Nord J Psychiat 60:360–364. 10.1080/08039480600937066 17050293

[50] Lavretsky H , Siddarth P , Kepe V , Ercoli LM , Miller KJ , Burggren AC *et al* (2009) Depression and anxiety symptoms are associated with cerebral FDDNP‐PET binding in middle‐aged and older nondemented adults. Am J Geriatr Psychiatry 17:493–502. 19472439 10.1097/jgp.0b013e3181953b82 PMC2709773

[51] Li JY , Englund E , Holton JL , Soulet D , Hagell P , Lees AJ *et al* (2008) Lewy bodies in grafted neurons in subjects with Parkinson's disease suggest host‐to‐graft disease propagation. Nat Med 14:501–503. 18391963 10.1038/nm1746

[52] Lopez OL , Becker JT , Sweet RA , Martin‐Sanchez FJ , Hamilton RL (2006) Lewy bodies in the amygdala increase risk for major depression in subjects with Alzheimer disease. Neurology 67:660–665. 16924019 10.1212/01.wnl.0000230161.28299.3c

[53] Luk KC , Kehm V , Carroll J , Zhang B , O'Brien P , Trojanowski JQ , Lee VM (2012) Pathological alpha‐synuclein transmission initiates Parkinson‐like neurodegeneration in nontransgenic mice. Science 338:949–953. 23161999 10.1126/science.1227157 PMC3552321

[54] Luthi A , Luscher C (2014) Pathological circuit function underlying addiction and anxiety disorders. Nat Neurosci 17:1635–1643. 25402855 10.1038/nn.3849

[55] Madsen K , Hasselbalch BJ , Frederiksen KS , Haahr ME , Gade A , Law I *et al* (2012) Lack of association between prior depressive episodes and cerebral [C‐11]PiB binding. Neurobiol Aging 33:2334–2342. 22192243 10.1016/j.neurobiolaging.2011.11.021

[56] Mattis PJ , Tang CC , Ma Y , Dhawan V , Eidelberg D (2011) Network correlates of the cognitive response to levodopa in Parkinson disease. Neurology 77:858–865. 21849641 10.1212/WNL.0b013e31822c6224 PMC3162641

[57] McKeith I , Cummings J (2005) Behavioural changes and psychological symptoms in dementia disorders. Lancet Neurol 4:735–742. 16239180 10.1016/S1474-4422(05)70219-2

[58] McKeith IG (2000) Clinical Lewy body syndromes. Ann N Y Acad Sci 920:1–8. 11193136 10.1111/j.1749-6632.2000.tb06898.x

[59] McKeith IG , Boeve BF , Dickson DW *et al* (2017) Diagnosis and management of dementia with Lewy bodies: Fourth consensus report of the DLB Consortium. Neurology 89:88–100. 28592453 10.1212/WNL.0000000000004058 PMC5496518

[60] McKeith IG , Dickson DW , Lowe J *et al* (2005) Diagnosis and management of dementia with Lewy bodies: third report of the DLB Consortium. Neurology 65:1863–1872. 16237129 10.1212/01.wnl.0000187889.17253.b1

[61] McKinlay A , Grace RC , Dalrymple‐Alford JC , Anderson T , Fink J , Roger D (2008) A profile of neuropsychiatric problems and their relationship to quality of life for Parkinson's disease patients without dementia. Parkinsonism Relat D 14(1):37–42. 10.1016/j.parkreldis.2007.05.009 17627863

[62] Menza M , Dobkin RD , Marin H , Mark MH , Gara M , Buyske S *et al* (2009) The impact of treatment of depression on quality of life, disability and relapse in patients with Parkinson's disease. Mov Disord 24:1325–1332. 19412944 10.1002/mds.22586 PMC2819360

[63] Meredith GE , Pennartz CM , Groenewegen HJ (1993) The cellular framework for chemical signalling in the nucleus accumbens. Prog Brain Res 99:3–24. 7906426 10.1016/s0079-6123(08)61335-7

[64] Meynen G , Van Stralen H , Smit JH , Kamphorst W , Swaab DF , Hoogendijk WJ (2010) Relation between neuritic plaques and depressive state in Alzheimer's disease. Acta Neuropsychiatr 22:14–20. 25384952 10.1111/j.1601-5215.2009.00423.x

[65] Meynen G , van Stralen H , Smit JH , Kamphorst W , Swaab DF , Hoogendijk WJG (2010) Relation between neuritic plaques and depressive state in Alzheimer's disease. Acta Neuropsychiatrica 22:14–20. 25384952 10.1111/j.1601-5215.2009.00423.x

[66] Montine TJ , Phelps CH , Beach TG *et al* (2012) National Institute on Aging‐Alzheimer's Association guidelines for the neuropathologic assessment of Alzheimer's disease: a practical approach. Acta Neuropathologica 123:1–11. 22101365 10.1007/s00401-011-0910-3 PMC3268003

[67] Mouton PR , Price DL , Walker LC (1997) Empirical assessment of synapse numbers in primate neocortex. J Neurosci Methods 75:119–126. 9288643 10.1016/s0165-0270(97)00058-7

[68] Nagano‐Saito A , Leyton M , Monchi O , Goldberg YK , He Y , Dagher A (2008) Dopamine depletion impairs frontostriatal functional connectivity during a set‐shifting task. J Neurosci 28:3697–3706. 18385328 10.1523/JNEUROSCI.3921-07.2008 PMC6671089

[69] Nelson AB , Kreitzer AC (2014) Reassessing models of basal ganglia function and dysfunction. Annu Rev Neurosci 37:117–135. 25032493 10.1146/annurev-neuro-071013-013916 PMC4416475

[70] Niethammer M , Tang CC , Ma Y , Mattis PJ , Ko JH , Dhawan V , Eidelberg D (2013) Parkinson's disease cognitive network correlates with caudate dopamine. NeuroImage 78:204–209. 23578575 10.1016/j.neuroimage.2013.03.070 PMC3672243

[71] Norris EH , Giasson BI , Lee VM (2004) Alpha‐synuclein: normal function and role in neurodegenerative diseases. Curr Top Dev Biol 60:17–54. 15094295 10.1016/S0070-2153(04)60002-0

[72] O'Brien JT , Colloby S , Fenwick J , Williams ED , Firbank M , Burn D *et al* (2004) Dopamine transporter loss visualized with FP‐CIT SPECT in the differential diagnosis of dementia with Lewy bodies. Arch Neurol 61:919–925. 15210531 10.1001/archneur.61.6.919

[73] Phan JA , Stokholm K , Zareba‐Paslawska J , Jakobsen S , Vang K , Gjedde A *et al* (2017) Early synaptic dysfunction induced by alpha‐synuclein in a rat model of Parkinson's disease. Sci Rep 7:6363. 28743955 10.1038/s41598-017-06724-9 PMC5526979

[74] Piggott MA , Marshall EF , Thomas N , Lloyd S , Court JA , Jaros E *et al* (1999) Striatal dopaminergic markers in dementia with Lewy bodies, Alzheimer's and Parkinson's diseases: rostrocaudal distribution. Brain 122:1449–1468. 10430831 10.1093/brain/122.8.1449

[75] Politis M , Wu K , Loane C , Turkheimer FE , Molloy S , Brooks DJ , Piccini P (2010) Depressive symptoms in PD correlate with higher 5‐HTT binding in raphe and limbic structures. Neurology 75:1920–1927. 21098407 10.1212/WNL.0b013e3181feb2ab

[76] Pont‐Sunyer C , Hotter A , Gaig C , Seppi K , Compta Y , Katzenschlager R *et al* (2015) The onset of nonmotor symptoms in Parkinson's disease (the ONSET PD study). Mov Disord Off J Move Disord Soc 30:229–237. 10.1002/mds.26077 25449044

[77] Pontone GM , Williams JR , Anderson KE , Chase G , Goldstein SA , Grill S *et al* (2009) Prevalence of anxiety disorders and anxiety subtypes in patients with Parkinson's disease. Mov Disord Off J Move Disord Soc 24:1333–1338. 10.1002/mds.22611 PMC2830642 19425086

[78] Rapp MA , Schnaider‐Beeri M , Grossman HT , Sano M , Perl DP , Purohit DP *et al* (2006) Increased hippocampal plaques and tangles in patients with Alzheimer disease with a lifetime history of major depression. Arch Gen Psychiatry 63:161–167. 16461859 10.1001/archpsyc.63.2.161

[79] Rapp MA , Schnaider‐Beeri M , Purohit DP , Perl DP , Haroutunian V , Sano M (2008) Increased neurofibrillary tangles in patients with Alzheimer disease with comorbid depression. Am J Geriatr Psychiatry 16:168–174. 18239198 10.1097/JGP.0b013e31816029ec

[80] Reyes JF , Olsson TT , Lamberts JT , Devine MJ , Kunath T , Brundin P (2015) A cell culture model for monitoring alpha‐synuclein cell‐to‐cell transfer. Neurobiol Dis 77:266–275. 25046995 10.1016/j.nbd.2014.07.003

[81] Ring HA , Serra‐Mestres J (2002) Neuropsychiatry of the basal ganglia. J Neurol Neurosurg Psychiatry 72:12–21. 11784818 10.1136/jnnp.72.1.12 PMC1737705

[82] Root DH , Melendez RI , Zaborszky L , Napier TC (2015) The ventral pallidum: Subregion‐specific functional anatomy and roles in motivated behaviors. Prog Neurogibol 130:29–70. 10.1016/j.pneurobio.2015.03.005 PMC4687907 25857550

[83] Ross GW , Petrovitch H , Abbott RD , Nelson J , Markesbery W , Davis D *et al* (2004) Parkinsonian signs and substantia nigra neuron density in decendents elders without PD. Ann Neurol 56:532–539. 15389895 10.1002/ana.20226

[84] Rosso G , Rigardetto S , Bogetto F , Maina G (2012) A randomized, single‐blind, comparison of duloxetine with bupropion in the treatment of SSRI‐resistant major depression. J Affect Disord 136:172–176. 21862138 10.1016/j.jad.2011.07.026

[85] Schaeffer E , Berg D (2017) Dopaminergic therapies for non‐motor symptoms in parkinson's disease. CNS Drugs 31:551–570. 28643183 10.1007/s40263-017-0450-z

[86] Schmidt HD , Famous KR , Pierce RC (2009) The limbic circuitry underlying cocaine seeking encompasses the PPTg/LDT. Eur J Neuorsci 30:1358–1369. 10.1111/j.1460-9568.2009.06904.x PMC2875792 19788581

[87] Schneider CA , Rasband WS , Eliceiri KW (2012) NIH Image to ImageJ: 25 years of image analysis. Nat Methods 9:671–675. 22930834 10.1038/nmeth.2089 PMC5554542

[88] Shiba M , Bower JH , Maraganore DM , McDonnell SK , Peterson BJ , Ahlskog JE *et al* (2000) Anxiety disorders and depressive disorders preceding Parkinson's disease: a case‐control study. Mov Disord Off J Move Disord Soc 15:669–677. 10.1002/1531-8257(200007)15:4<669::aid-mds1011>3.0.co;2-5 10928577

[89] Shimozawa A , Ono M , Takahara D , Tarutani A , Imura S , Masuda‐Suzukake M *et al* (2017) Propagation of pathological alpha‐synuclein in marmoset brain. Acta Neuropathol Commun 5:12. 28148299 10.1186/s40478-017-0413-0 PMC5289012

[90] Skapinakis P , Bakola E , Salanti G , Lewis G , Kyritsis AP , Mavreas V (2010) Efficacy and acceptability of selective serotonin reuptake inhibitors for the treatment of depression in Parkinson's disease: a systematic review and meta‐analysis of randomized controlled trials. BMC Neurol 10:49. 20565960 10.1186/1471-2377-10-49 PMC2903535

[91] Spillantini MG , Goedert M (2016) Synucleinopathies: past, present and future. Neuropathol Appl Neurobiol 42:3–5. 26819143 10.1111/nan.12311

[92] Stahl SM , Zhang L , Damatarca C , Grady M (2003) Brain circuits determine destiny in depression: a novel approach to the psychopharmacology of wakefulness, fatigue, and executive dysfunction in major depressive disorder. J Clin Psychiatry 64(Suppl 14):6–17. 14658930

[93] Syed A , Chatfield M , Matthews F , Harrison P , Brayne C , Esiri MM (2005) Depression in the elderly: pathological study of raphe and locus ceruleus. Neuropathol Appl Neurobiol 31:405–413. 16008824 10.1111/j.1365-2990.2005.00662.x

[94] Tsopelas C , Stewart R , Savva GM , Brayne C , Ince P , Thomas A *et al* (2011) Neuropathological correlates of late‐life depression in older people. Brit J Psychiat 198:109–114. 10.1192/bjp.bp.110.078816 21282780

[95] Tye KM , Mirzabekov JJ , Warden MR *et al* (2013) Dopamine neurons modulate neural encoding and expression of depression‐related behaviour. Nature 493:537‐+. 23235822 10.1038/nature11740 PMC4160519

[96] Vermeiren Y , De Deyn PP (2017) Targeting the norepinephrinergic system in Parkinson's disease and related disorders: the locus coeruleus story. Neurochem Int 102:22–32. 27899296 10.1016/j.neuint.2016.11.009

[97] Vermeiren Y , Van Dam D , Aerts T , Engelborghs S , De Deyn PP (2014) Monoaminergic neurotransmitter alterations in postmortem brain regions of depressed and aggressive patients with Alzheimer's disease. Neurobiol Aging 35:2691–2700. 24997673 10.1016/j.neurobiolaging.2014.05.031

[98] Vermeiren Y , Van Dam D , Aerts T , Engelborghs S , Martin JJ , De Deyn PP (2015) The monoaminergic footprint of depression and psychosis in dementia with Lewy bodies compared to Alzheimer's disease. Alzheimers Res Ther 7:7. 25717350 10.1186/s13195-014-0090-1 PMC4339739

[99] Walker L , McAleese KE , Thomas AJ *et al* (2015) Neuropathologically mixed Alzheimer's and Lewy body disease: burden of pathological protein aggregates differs between clinical phenotypes. Acta Neuropathologica 129:729–748. 25758940 10.1007/s00401-015-1406-3

[100] Warren JD , Rohrer JD , Schott JM , Fox NC , Hardy J , Rossor MN (2013) Molecular nexopathies: a new paradigm of neurodegenerative disease. Trends Neurosci 36:561–569. 23876425 10.1016/j.tins.2013.06.007 PMC3794159

[101] Watson R , Blamire AM , O'Brien JT (2009) Magnetic resonance imaging in lewy body dementias. Dement Geriatr Cogn Disord 28:493–506. 19996594 10.1159/000264614

[102] Wen MC , Chan LL , Tan LC , Tan EK (2016) Depression, anxiety, and apathy in Parkinson's disease: insights from neuroimaging studies. Eur J Neurol 23:1001–1119. 27141858 10.1111/ene.13002 PMC5084819

[103] Wertman E , Speedie L , Shemesh Z , Gilon D , Raphael M , Stessman J (1993) Cognitive disturbances in parkinsonian‐patients with depression. Neuropsychiatry Neuropsychol Behav Neurol 6:31–37.

[104] Wilson RS , Schneider JA , Bienias JL , Arnold SE , Evans DA , Bennett DA (2003) Depressive symptoms, clinical AD, and cortical plaques and tangles in older persons. Neurology 61:1102–1107. 14581672 10.1212/01.wnl.0000092914.04345.97

[105] Witt K , Daniels C , Herzog J , Lorenz D , Volkmann J , Reiff J *et al* (2006) Differential effects of L‐dopa and subthalamic stimulation on depressive symptoms and hedonic tone in Parkinson's disease. J Neuropsychiatry Clin Neurosci 18:397–401. 16963590 10.1176/jnp.2006.18.3.397

[106] Wu KY , Hsiao IT , Chen CS , Chen CH , Hsieh CJ , Wai YY *et al* (2014) Increased brain amyloid deposition in patients with a lifetime history of major depression: evidenced on 18F‐florbetapir (AV‐45/Amyvid) positron emission tomography. Eur J Nucl Med Mol Imaging 41:714–722. 24233127 10.1007/s00259-013-2627-0

[107] Yavich L , Oksman M , Tanila H , Kerokoski P , Hiltunen M , van Groen T *et al* (2005) Locomotor activity and evoked dopamine release are reduced in mice overexpressing A30P‐mutated human alpha‐synuclein. Neurobiol Dis 20:303–313. 16242637 10.1016/j.nbd.2005.03.010

