# Supplementary figures and images for: Degeneration of dopaminergic circuitry influences depressive symptoms in Lewy body disorders
Source: Brain Pathol. 2019 Jan 29;29(4):544–57. doi: 10.1111/bpa.12697 (PMC6767514; doi:10.1111/bpa.12697)

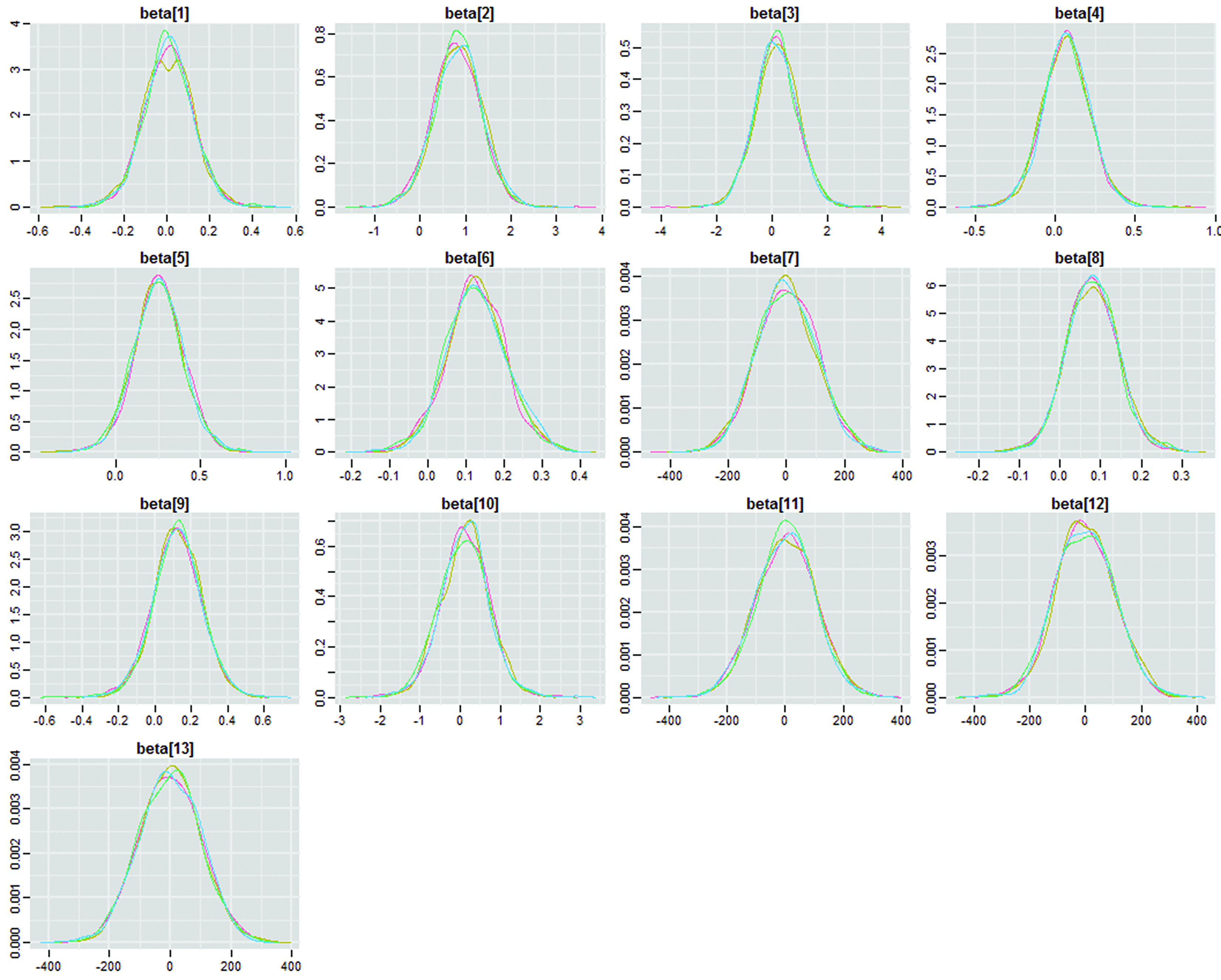

Supplement: Supplementary file 3 — Figure S3. Density plot for the SEM coefficients for the full model for deposition of α‐synuclein. Distributions for the coefficients beta(2), beta(5), beta(6) and beta(8) do not include zero, suggesting significant pathways between the two brain compartments. [file BPA-29-544-s006.tif]

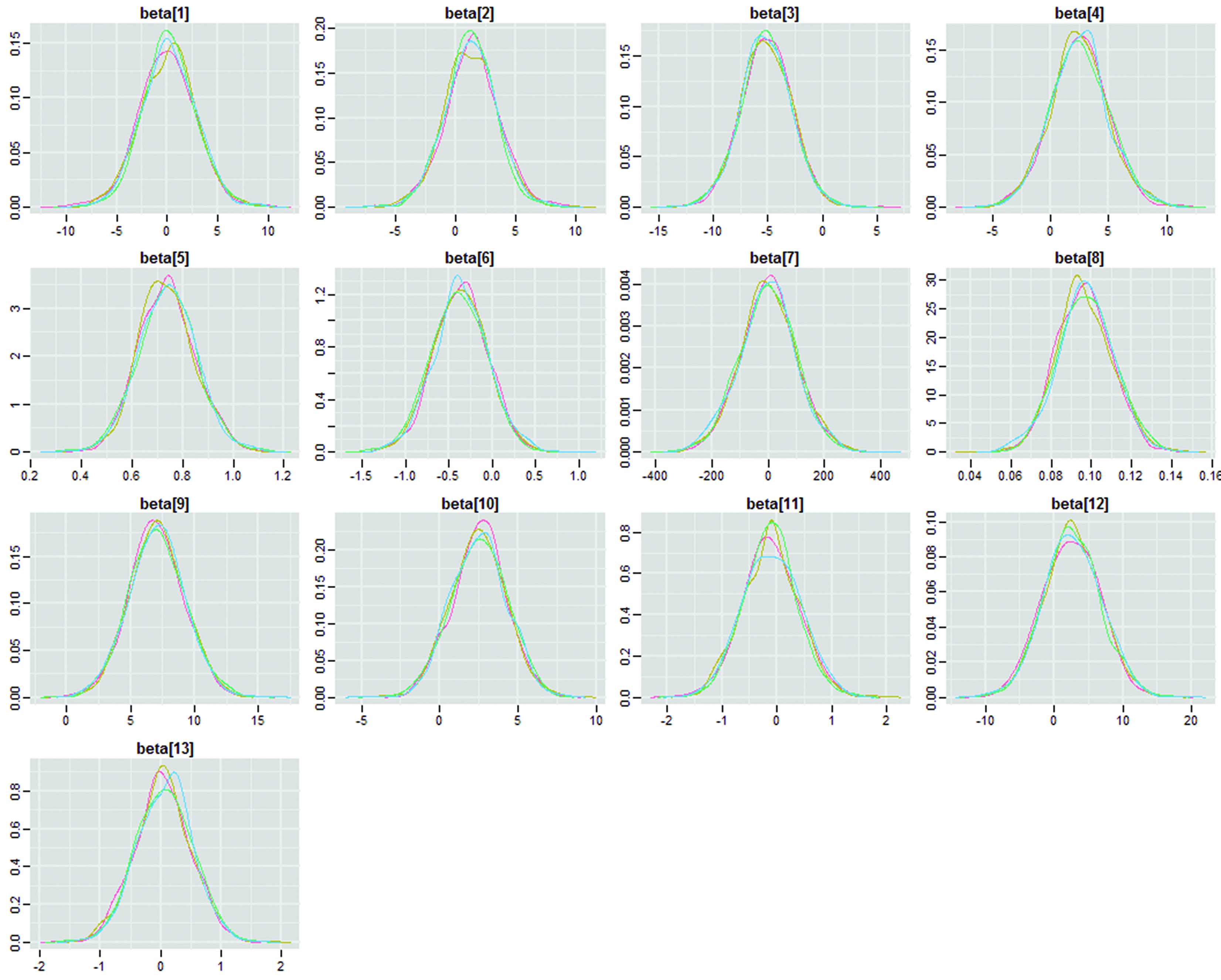

Supplement: Supplementary file 4 — Figure S4. Density plot for the SEM coefficients for the full model for deposition of tau protein. Distributions for the coefficients beta(3), beta(8) and beta(9) do not include zero suggesting significant pathways between the two brain compartments. [file BPA-29-544-s008.tif]

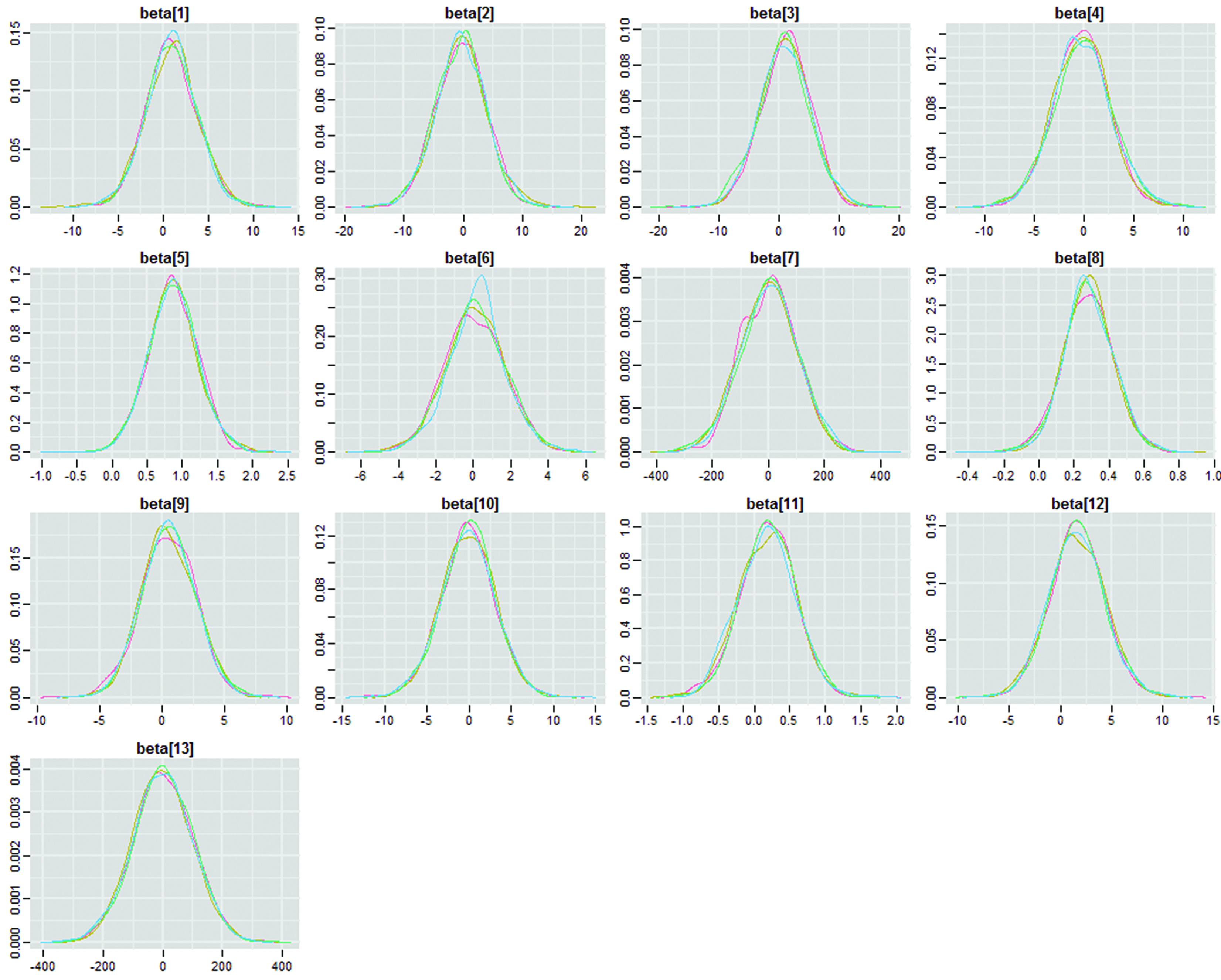

Supplement: Supplementary file 5 — Figure S5. Density plot for the SEM coefficients for the full model for deposition of amyloid beta. Distributions for the coefficients beta(5) and beta(8) suggesting significant pathways between the two brain compartments. [file BPA-29-544-s001.tif]
